# Fast Measurements with MOX Sensors: A Least-Squares Approach to Blind Deconvolution

**DOI:** 10.3390/s19184029

**Published:** 2019-09-18

**Authors:** Dominique Martinez, Javier Burgués, Santiago Marco

**Affiliations:** 1 Laboratoire Lorrain de Recherche en Informatique et ses Applications (LORIA), CNRS, INRIA, 54506 Vandoeuvre-lès-Nancy, France; 2 Institute for Bioengineering of Catalonia (IBEC), The Barcelona Institute of Science and Technology, Baldiri Reixac 10-12, 08028 Barcelona, Spain; jburgues@ibecbarcelona.eu (J.B.); santiago.marco@ub.edu (S.M.); 3 Department of Electronics and Biomedical Engineering, Universitat de Barcelona, Marti i Franqués 1, 08028 Barcelona, Spain

**Keywords:** MOX sensors, blind deconvolution, blind identification, least-squares, turbulent plumes

## Abstract

Metal oxide (MOX) sensors are widely used for chemical sensing due to their low cost, miniaturization, low power consumption and durability. Yet, getting instantaneous measurements of fluctuating gas concentration in turbulent plumes is not possible due to their slow response time. In this paper, we show that the slow response of MOX sensors can be compensated by deconvolution, provided that an invertible, parametrized, sensor model is available. We consider a nonlinear, first-order dynamic model that is mathematically tractable for MOX identification and deconvolution. By transforming the sensor signal in the log-domain, the system becomes linear in the parameters and these can be estimated by the least-squares techniques. Moreover, we use the MOX diversity in a sensor array to avoid training with a supervised signal. The information provided by two (or more) sensors, exposed to the same flow but responding with different dynamics, is exploited to recover the ground truth signal (gas input). This approach is known as blind deconvolution. We demonstrate its efficiency on MOX sensors recorded in turbulent plumes. The reconstructed signal is similar to the one obtained with a fast photo-ionization detector (PID). The technique is thus relevant to track a fast-changing gas concentration with MOX sensors, resulting in a compensated response time comparable to that of a PID.

## 1. Introduction

Air pollution is a major problem affecting the health of people, leading to 4 million deaths each year [1]. Moreover, half of the worldwide population lives in cities where pollutant emission sources can vary sharply in location and time [2]. Mapping spatiotemporal air pollution in urban cities at a fine scale is thus of crucial importance. The large number of vehicles circulating in city streets, e.g., garbage trucks, buses or taxis, could be used for this purpose if they were equipped with on-board chemical sensors [3]. A prerequisite, however, is that low-cost and fast-response sensors are available. When an air quality map is built from measurements acquired in motion, the sensor has to be very fast or else the resulting map will be blurred [4]. This constraint is particularly true when the mobile sensing platform is a fast moving vehicle.

Certainly, the fastest chemical sensors are biological sensors. The olfactory response of insect antennae is recorded in the form of electroantennograms (EAGs) that have a response time of tens of ms [5]. Previously, EAGs have been used as chemical sensors on mobile (robotic) platforms [6]. Yet, the biological preparation is complex, and its lifetime is too limited to be used routinely. In contrast, artificial sensors are more robust and easy to use. Photo-ionization detectors (PIDs) can compete with EAGs in terms of response time. Unfortunately, they are too bulky and expensive to be deployed on a large fleet of existing vehicles or in platforms with limited payload such as nano-drones [7]. In contrast, metal oxide (MOX) gas sensors are miniaturized and inexpensive, with a cost of ~10 USD, which is compatible with a wide-scale deployment. They are also sensitive to the volatile organic compounds (VOCs) relevant to environmental monitoring. Yet, they have a slow response time of tens of seconds, which prevents their use on fast moving vehicles.

**Figure 1:**
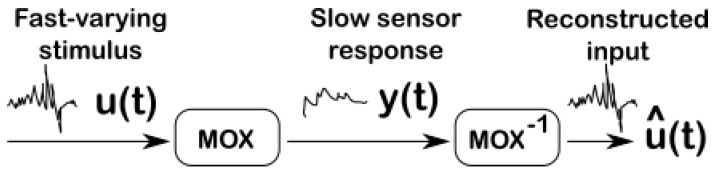
Deconvolution of metal oxide (MOX) gas sensors sensors. The deconvolution operation consists of reconstructing the fast-varying concentration u(t) encountered in turbulent plumes by inverting the low-pass filtering effect of MOX sensors.

The slow response time of MOX sensors is a consequence of a slow rise time upon gas exposure and an even slower recovery time upon gas removal. The slow recovery time can be compensated by using a multi-chamber system [8]. Yet, the required additional electrovalves and fluidic components increase the overall cost, size, weight and power consumption of the system. An alternative approach to improve both the rise and recovery time without resorting to external components is by deconvolution. The idea behind deconvolution is that if one is able to identify a parametrized, invertible, model of the sensor dynamics, we could invert it to uncover the original fast-varying stimulus u(t) from the slow-varying sensor signal y(t) (see Figure 1). Previous deconvolution approaches of MOX sensors [4,9] suffer from some drawbacks however. First, they consider a linear sensor model without taking into account the intrinsic nonlinearity of MOX sensors [10]. Second, the linear model is identified and validated under unrealistic conditions with a large gas chamber affecting the sensor dynamics and/or concentration steps of a long duration (several minutes) that are not representative of a real turbulent plume.

Here, we propose a combined identification/deconvolution approach based on a nonlinear model of MOX sensors and realistic stimulation conditions, i.e., natural odor plumes. We consider a nonlinear, first-order dynamic model that is mathematically tractable for MOX identification and deconvolution. By transforming the sensor signal in the log-domain, the system becomes linear in the parameters and these can be estimated by the least-squares techniques. The identification can be performed in a supervised way from a training signal; that is, a reference of the fluctuating gas concentration in turbulent plumes provided by a fast photo-ionization detector (PID). However, this supervised approach is unsuitable in certain applications as retraining with the PID must be performed regularly to adapt to changing sensor characteristics (e.g., to overcome the drift due to contamination or poisoning of the sensing material) and environmental conditions (e.g., changes in humidity and temperature). In order to avoid the use of a PID, we exploit the information provided by two MOX sensors employed in parallel and use each one as a reference of the other. This approach is known as blind deconvolution. Blind deconvolution has been performed in the past for linear thermocouple sensors, see e.g., [11,12], but not for nonlinear MOX sensors. Here, we demonstrate its efficiency in a challenging scenario in which the goal is to track the fast concentration fluctuations of a turbulent plume (several kHz) by using two MOX sensors with a nominal bandwidth below 1 Hz.

The rest of the paper is organized as follows. In Section 2, we propose a nonlinear first-order dynamic model of MOX sensors that is tractable for identification and deconvolution. Although the model has a limited number of parameters, identifying them directly leads to a difficult nonlinear estimation problem. In Section 3, we derive a linearization technique so that ordinary least-squares methods can be employed. The supervised method which requires prior training with a fast PID is presented in Section 4 and the blind method which exploits the information from two MOX sensors is detailed in Section 5. This technique is generalized in Section 6 to more than two sensors and more complex sensor dynamics. Finally, the main conclusions are presented in Section 7.

Throughout this paper, the following notation is used. Scalar variables are indicated by lowercase letters, e.g., x, and vectors as boldface lowercase letters, e.g., x. Matrices are denoted by boldface uppercase letters, e.g., X. The prime notation denotes the logarithm of a variable or parameter, e.g., x′ is logx, and a hat denotes an estimate of a parameter, e.g., $\hat{x}$.

## 2. A Simple Nonlinear Model of MOX Sensors

**Figure 2:**
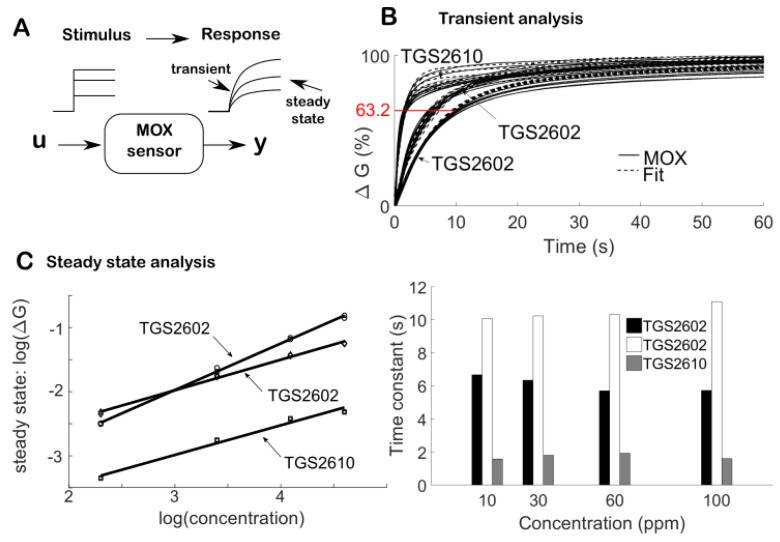
**A** The step response of MOX sensors. The sensor conductance G is measured over time in response to ethanol steps at different concentrations. See Appendix A for details of the experimental setup. **B** Transient response analysis. Top) The conductance change ΔG over time in response to a step-like stimulus. The transient is well-fitted by a sum of two exponential functions that represent the adsorption process of the chemical compound onto the sensing element. Here, the time constant corresponds to the time at which the MOX reaches (1 − 1/e) × 100% ≈ 63.2% of the steady state value. Bottom) The time constant (10.4 ± 0.46 and 6.1 ± 0.47 for the 2 × TGS2602, 1.7 ± 0.19 for the TGS2610 in sec.) depends on the sensor type but is relatively independent on input intensity (ethanol concentration). **C** Steady state response analysis. The steady-state conductance is well-described by a power law function.

The objective of this section is to develop a simple model of a MOX sensor that allows for identification and deconvolution. We first consider a MOX sensor as a linear dynamical system with impulse response h(t). The response x(t) to any input signal—fluctuating odor concentration u(t)—is given by the convolution x(t)=h(t)∗u(t), where ∗ is the convolution operator. The main purpose of the deconvolution is to uncover the incoming signal u(t) by inverting the convolution process; i.e., $U\left(f\right)=X\left(f\right)H{\left(f\right)}^{-1}$ in the frequency domain. Identifying the impulse response is thus a prerequisite to deconvolution. Although finding the response to an impulse may be possible for some particular sensors—e.g., by exciting a visual sensor with a pulse-laser—it is experimentally difficult to stimulate a gas sensor with a brief chemical input. Rather, we estimate the dynamics of the sensor from its response to step-like stimuli (Figure 2A and Appendix A). The impulse response is then obtained as the derivative of the step response.

We stimulate three MOX sensors (2 × TGS2602, 1 × TGS2610) in several steps, using ethanol at various concentrations (10, 30, 60 and 100 ppm) (see Appendix A for experimental details). These sensors are commercialized with a cap that has a flame-proof stainless steel gauze. The sensor cap introduces an additional low-pass filtering in the response due to the time required for the gas to diffuse through the gauze and reach the MOX surface. We therefore removed the cap to improve the MOX response time, e.g., as in [13]. The sensing elements are then encased in miniaturized gas chambers (volume of 0.32 mL per sensor) acting as protective shells. The 3D-printed fluidic design is described in [14]. The individual chambers are filled by the inlet flow in 75 ms.

The step response of these modified MOX sensors is shown in Figure 2B (top). It is well fitted using exponential models with a limited number of terms; merely one or two in agreement with previous studies [15]. With two exponential terms, the deconvolution filter in the time domain requires second-order derivatives of the signal (see Appendix A). As these high-order derivatives are extremely noisy to compute numerically, we chose to model the response to a unit step as a single exponential; that is 1−e−tτ with τ the time constant of the sensor. This model is reasonable given that the time constant depends on the sensor type but not on the ethanol concentration, at least in the range tested (Figure 2B, bottom). The continuous-time deconvolution filter obeys the linear differential equation (Appendix A):

(1) $$u\left(t\right)=x\left(t\right)+\;\tau \frac{dx}{dt}$$

The original signal u(t) can be reconstructed from Equation (1) if the time constant τ of the sensor is known and the derivative of the sensor output is available. In practice, the sensor output is noisy and the derivative is difficult to estimate accurately. To avoid computing signal derivatives, we integrate Equation (1) within the sampling interval Δt. This allows us to provide a discrete-time implementation of the deconvolution filter as

(2) $$x_{k+1}=a\;x_{k}+\left(1-a\right)\;u_{k}$$

with a=e−Δtτ. Typically, for MOX sensors we have τ≫Δt (τ and Δt are in the order of second and millisecond, respectively) so that a→1. In our experimental data (Figure 1B, bottom), the time constant was τ (in s) = 10.4 ± 0.46 and 6.1 ± 0.47 for the two TGS2602, and τ = 1.7 ± 0.19 for the TGS2610. Given that Δt=1 ms, we have a = 0.9999 and 0.9998 for the two TGS2602, and a = 0.9994 for the TGS2610.

**Figure 3:**
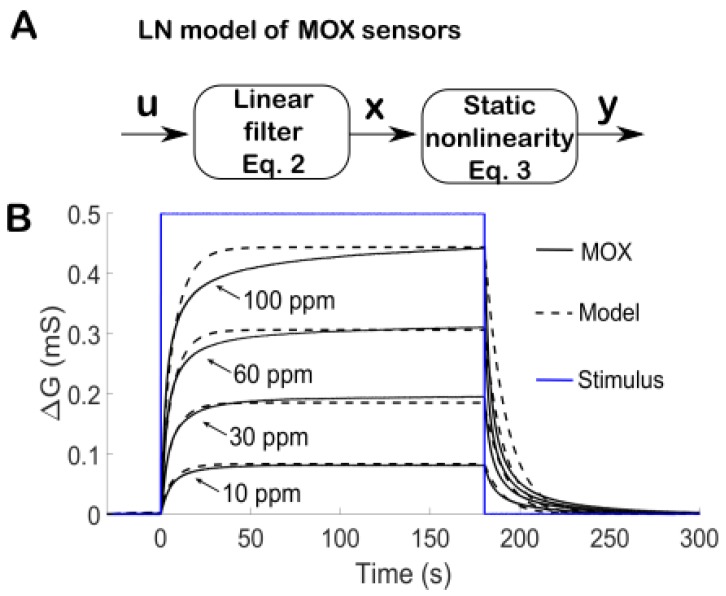
**A** The LN model of MOX sensors described by a cascade of a linear filter (Equation (2)) and a static power-law nonlinearity (Equation (3)). **B** Comparisons with experimental data. Simulation of the LN model (dashed black curve with a = 0.9999, α = 0.0157, r = 0.7256) and comparison with real data (plain black curve) in response to a step-like stimulation of ethanol (blue curve) at different concentrations.

So far, we have considered a MOX sensor as a linear time-invariant system. If it is a linear system, it should obey the principle of homogeneity, that is, the steady state response should be proportional to the input intensity. Yet, the steady state of MOX sensors follows a power law [10], that is

(3) $$y_{k}=\alpha \;x_{k}{}^{r}$$

where α the r are parameters that depend on the sensor type (see Figure 2C). Thus, MOX sensors are not linear and cannot be identified uniquely by an impulse response. Here, we model MOX sensors as a linear–nonlinear (LN) system (Figure 3A), which consists of a nonlinear cascade of a 1st-order low-pass filter given by Equation (2) and a static nonlinearity given by Equation (3).

Figure 3B compares the LN model to real data (TGS2610) in response to step-like stimuli of ethanol. We note that our non-linear model with a single time constant fits reasonably well, no matter if the conductance increases or decreases. Thus, in our study there is no need to have separate models for the two phases. Yet, there is a mismatch during the transient that increases with concentration (see at 60 and 100 ppm). This discrepancy comes from the simple dynamics parametrized by a single time constant (Equation (1)). A better match would have been obtained by fitting the transient with two exponential terms (Figure 2B top). Yet, Equation (1) would then involve additional parameters as well as the second-order derivative of the signal, which is extremely noisy to compute numerically. To limit the number of parameters and the noise, we made the model as simple as possible, but not simpler. As shown later, this model is quite effective in its goals of identification and deconvolution.

## 3. A Linearization Technique from Logarithmic Transformation

Our LN model of MOX sensors can be seen as a state-space system, with Equations (2) and (3) being respectively the state transition and observation equations. Note that both equations are non-linear due to the product $a\;x_{k}$ between the parameter and the state in the state transition equation, and due to the power law $\alpha \;x_{k}{}^{r}$ in the observation equation. The extended Kalman filter (EKF) could potentially be applied to estimate the three parameters (a, α, r) and state $x_{k}$ from a known input $u_{k}$. The EKF is the nonlinear version of the Kalman filter, which linearizes the model around the current estimate. Yet, it turned out that the EKF suffers from convergence problems owing to its linearization when the initial estimate of the state is wrong. In the following, we propose a technique of linearization so that the state variable $x_{k}$ can be eliminated and the system becomes linear in the parameters (a, α, r) and these can be identified by ordinary least squares techniques.

First we note that a logarithm transformation of the sensor output allows us to linearize the power law (Equation (3)) in our LN model, such that

(4) $$\log[y_{k}]\;=\;\log[\alpha ]\;+\;r\;\log[x_{k}]$$

This logarithm transformation of the steady state is illustrated in Figure 2C. Moreover, because the logarithm is a concave function, we have

**Figure 4:**
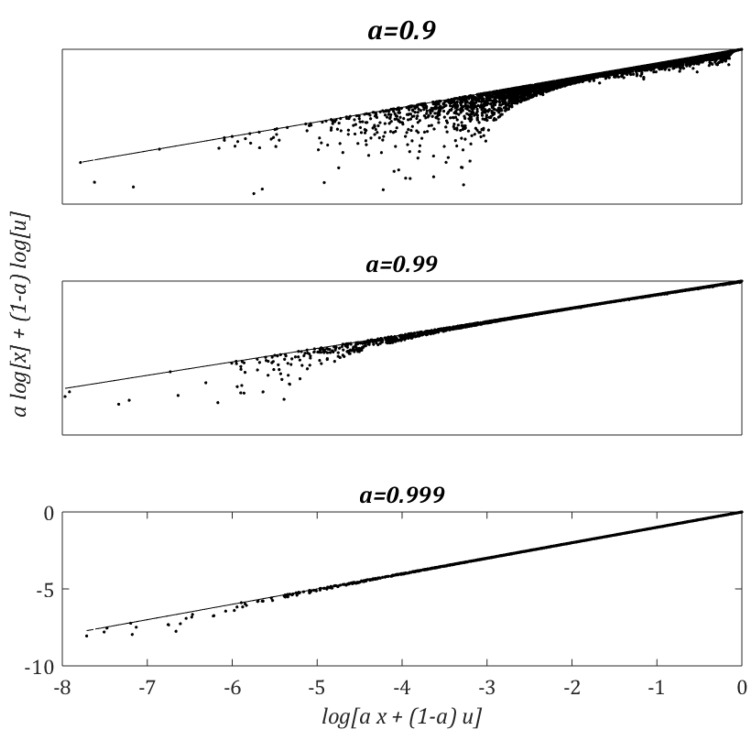
Numerical evaluation of the lower bound (Equation (5)) for different values of a. The line in the graphs corresponds to equality (Equation (6)). For a given value of a, the variables *x* and *u* are sampled randomly in the unit interval, each point corresponding to a particular realization.

(5) log[a x+(1−a) u] ≥ a log[x] + (1 − a) log[u]

for any a∈[0,1]. Figure 4 compares the lower bound given by Equation (5) for different values of a. We note that the inequality in (5) becomes an equality when a→1, which is a condition typical of MOX sensors (e.g., a=0.999 when τ=1 s and Δt=1 ms). We can therefore rewrite (Equation (2)) as

(6) $$\log[x_{k+1}]\;\approx \;a\;\log[x_{k}]+(1-a)\;\log[u_{k}]$$

and our model of MOX sensors (Equations (2) and (3)) becomes

(7) $$x_{k+1}^{\prime }=\;a\;x_{k}^{\prime }+\;(1-a)\;u_{k}^{\prime }$$

$$y_{k}^{\prime }=\alpha^{\prime }+\;rx_{k}^{\prime }$$

with the change of variables $x^{\prime }=\log\left[x\right]$, $u^{\prime }=\log\left[u\right]$, $y^{\prime }=\log\left[y\right]$ and $\alpha^{\prime }=\log\left[\alpha \right]$. In the sequel, the prime notation denotes the logarithm of variables or parameters. The transformed state variable $x^{\prime }$ can be further eliminated by rewriting Equation (7) as

(8) $$y_{k+1}^{\prime }\;=\;a\;y_{k}^{\prime }+b\;u_{k}^{\prime }\;+c$$

with b=r(1−a) and $c=\alpha^{\prime }\left(1-a\right)$. Note that Equation (8) is now linear in the parameters and these can be identified by ordinary least squares techniques.

## 4. Supervised Identification/Deconvolution in the Log-Domain

**Figure 5:**
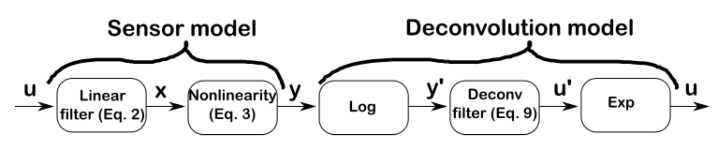
Deconvolution in the log-domain based on the sensor model Equations (2) and (3). The deconvolution operation consists of taking the logarithm of the MOX signal, processing this log signal with a linear filter (Equation (9)), and recovering the output via an exponential function.

Figure 5 summarizes the deconvolution operation which consists of taking the logarithm of the MOX signal, processing this log signal with a linear filter, and recovering the output via an exponential function. The deconvolution filter in the log-domain follows from Equation (8) as

(9) $$u_{k}^{\prime }\;=\;w_{1}\;y_{k+1}^{\prime }+w_{2}\;y_{k}^{\prime }+w_{3}$$

with filter coefficients $w_{1}=1/\left[r\left(1-a\right)\right]$, $w_{2}=-a/\left[r\left(1-a\right)\right]$ and $w_{3}=-\alpha^{\prime }/r$.

The original signal $u_{k}=e^{u_{k}^{\prime }}$ can be reconstructed from Equation (9) if the filter coefficients are known.

We consider that samples of $u_{k}$ are available as a supervised signal for estimating $w_{1},\;w_{2},\;w_{3}$. Let us denote U′=($u\prime_{1},u\prime_{2},\cdots ,u\prime_{n}{)}^{T}$ the *n* × 1 training vector. Equation (9) can be rewritten in matrix form as

(10) $$U\prime \;=\;Y^{\prime }\;w$$

with w=($w_{1},w_{2},w_{3}{)}^{T}$ the 3 × 1 vector of filter coefficients and $Y^{\prime }$ the following *n* × 3 matrix

(11) $$Y^{\prime }=\left(\begin{array}{c} \begin{array}{ccc} y_{2}^{\prime }\; & y_{1}^{\prime } & 1\\ \vdots & \vdots & \vdots \\ y_{k}^{\prime } & y_{k-1}^{\prime } & 1 \end{array}\\ \begin{array}{ccc} \vdots & \vdots & \vdots \\ \vdots & \vdots & \vdots \\ y_{n+1}^{\prime } & y_{n}^{\prime } & 1 \end{array} \end{array}\right)$$

The filter coefficients are estimated so as to minimize the least square error defined by

(12) $$E\;=\;{\left\|Y^{\prime }\;w-U^{\prime }\right\|}^{2}$$

The solution is given by the pseudo-inverse, that is

(13) $$\hat{w}\;=\;(Y^{\prime T}Y^{\prime }{)}^{-1}Y^{\prime T}\;U^{\prime }$$

and the model parameters are identified from the filter coefficients as

(14) $$\{\begin{array}{c} \hat{a}=-{\hat{w}}_{2}/{\hat{w}}_{1}\left(\hat{\tau }=-\Delta t/\log\hat{a}\right)\;\\ \hat{r}=1/\left({\hat{w}}_{1}+{\hat{w}}_{2}\right)\\ \hat{\alpha }=e^{-\hat{r}{\hat{w}}_{3}} \end{array}$$

**Figure 6:**
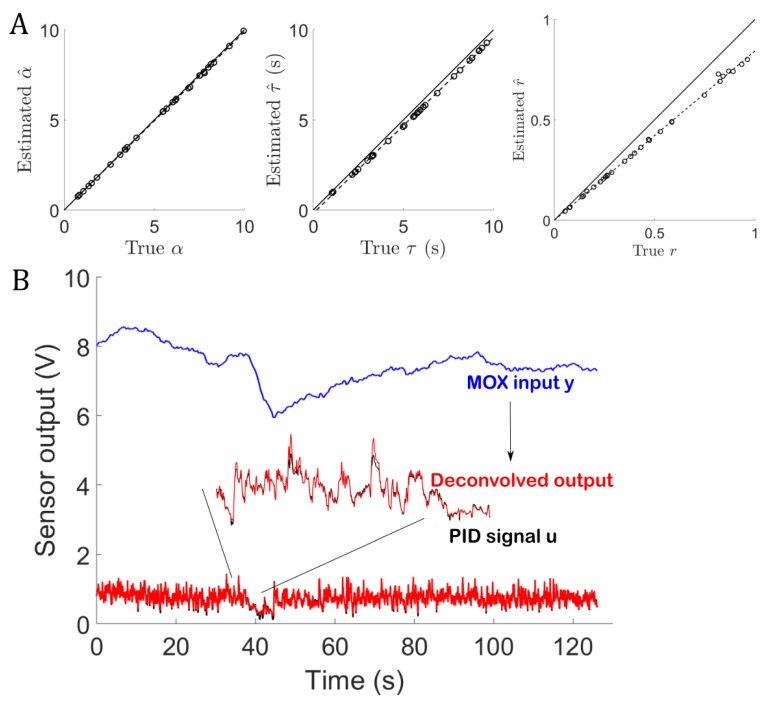
Supervised identification/deconvolution with a simulated MOX sensor. **A** Least-squares identification using Equations (13) and (14) (*n* = 30 trials). For each trial, the model parameters τ, r, α are randomly generated within (0,10 s), (0,1) and (0,10), respectively. **B** Deconvolution of the MOX output in the worst-case scenario: τ=10s, r=1, α=10 (maximum estimation bias in A).

To validate the identification method, we recorded the response of a PID (miniPiD 201 A from Aurora Scientific with a response time of a few ms) in a turbulent plume of ethanol. The PID was previously calibrated with ethanol in the range 0–100 ppm, so that its output over time is a good approximation of fluctuating ethanol concentration. We therefore consider the PID signal as $u_{k}$ (ground truth). We first performed experiments on a simulated MOX sensor based on Equations (2) and (3) with different parameter values (τ, r, α) randomly generated. Figure 6A compares the estimated parameters $(\hat{\tau },\;\hat{r},\;\hat{\alpha }$) obtained from Equations (13) and (14) to the true values used to simulate the MOX sensor. We found that our least-squares approach generates biased parameter estimates: $\hat{\tau }=0.97\;\tau ,\;\hat{\alpha }=0.99\;\alpha$ and $\hat{r}=0.84\;r$. The bias results from the approximation (Equation (6)) was used to linearize the model. Although it would be easy to compensate the estimation bias as it is systematic and linear (see Figure 6A), it is not necessary because (i) the bias is relatively small and (ii) it does not prevent recovery of the original PID signal $u_{k}$ (see Figure 6B).

**Figure 7:**
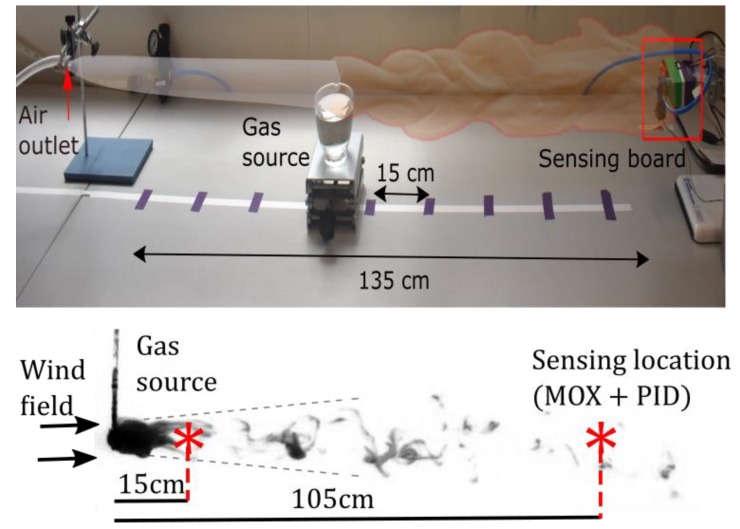
Generation of ethanol plumes in an open environment. The test bench uses a pressurized air outlet (6.3 mm radius, 20 L/min) and a vessel (5 cm radius) filled with 200 mL of ethanol (gas source). The sensing board, which consists of MOX sensors and a fast PID, was placed at 15 cm and 105 cm from the gas source.

**Figure 8:**
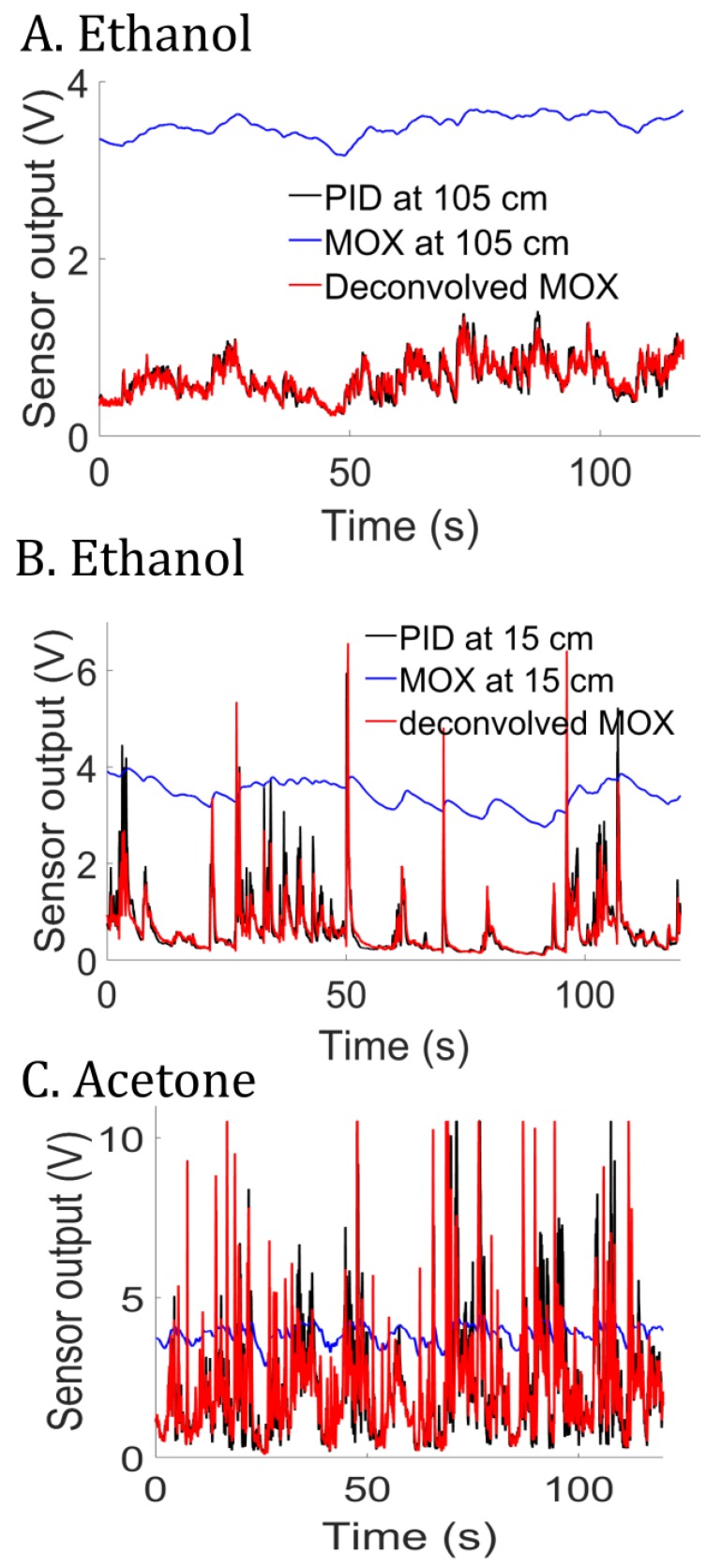
Supervised deconvolution experiments with a real MOX sensor. **A** Deconvolved MOX at 105 cm from the gas source (ethanol). **B** Deconvolved MOX at 15 cm from the gas source (ethanol). **C** Same as in B but with acetone as the gas source.

We further tested our least-squares method on a real MOX sensor (TGS2610) mounted with the miniPID in a small housing chamber (volume of 0.32 mL). Given the close proximity between the two sensors, we can reasonably assume that the MOX and the PID observe the same signal $u_{k}$. We generated ethanol plumes in an open environment as described in [14] and recorded the MOX and the PID at 15 cm and 105 cm from the source (Figure 7). The MOX output was filtered prior to identification to eliminate noise (5th-order Butterworth low-pass filter with 20 Hz cutting frequency). Figure 8A,B shows the deconvolved output of the MOX. We note that the deconvolution process leads to a signal that is similar to the PID in the two conditions (close and far from the source). The time constant estimated for the MOX is $\hat{\tau }=2.47$ s, irrespective of the recording location (15 or 105 cm). Moreover, the deconvolution approach is not limited to ethanol (see result with acetone in Figure 8C).

## 5. Blind Deconvolution Using Two Sensors

In the previous section, the system was identified by using a training sequence $u_{k}$ (PID signal) so as to have the deconvolved MOX as close as possible to the PID. In this section, we provide a blind technique for estimating the filter coefficients directly from the response of two MOX sensors, thereby avoiding the cost of training the system with a PID. In blind deconvolution, we consider that $u_{k}$ is unknown (the PID signal is not used). Instead, we exploit the information provided by two MOX sensors. The MOX sensors are both assumed to follow the NL model (Equations (2) and (3)) with unknown but different parameters. Denoting subscript 1 and 2 for sensor 1 and 2, respectively, the deconvolution filters in the log-domain follow from Equation (9) as

(15) $$u_{1,k}^{\prime }\;=\;w_{11}\;y_{1,k+1}^{\prime }+w_{12}\;y_{1,k}^{\prime }+w_{13}$$

$$u_{2,k}^{\prime }\;=\;w_{21}\;y_{2,k+1}^{\prime }+w_{22}\;y_{2,k}^{\prime }+w_{23}$$

with filter coefficients $w_{11}=1/\left[r_{1}\left(1-a_{1}\right)\right]$, $w_{12}=-a_{1}/\left[r_{1}\left(1-a_{1}\right)\right]$ and $w_{13}=-{\alpha_{1}}^{\prime }/r_{1}$ for sensor 1 and $w_{21}=1/\left[r_{2}\left(1-a_{2}\right)\right]$, $w_{22}=-a_{2}/\left[r_{2}\left(1-a_{2}\right)\right]$ and $w_{23}=-{\alpha_{2}}^{\prime }/r_{2}$ for sensor 2.

**Figure 9:**
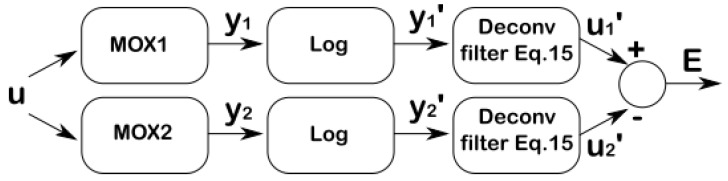
Blind deconvolution with two MOX sensors. The deconvolved outputs $u_{1}^{\prime }$ and $u_{2}^{\prime }$ should be similar as both sensors are excited by the same input u. The original input can be reconstructed as $\left(e^{u_{1}^{\prime }}+e^{u_{2}^{\prime }}\right)/2$.

As both sensors are excited by the same input $u_{k}$, the deconvolved outputs $u_{1,k}^{\prime }$ and $u_{2,k}^{\prime }$ should be similar (see Figure 9). Thus, we performed blind identification of the system (Equation (15)) by minimizing the difference between $u_{1,k}^{\prime }$ and $u_{2,k}^{\prime }$ given by the least-squares error

(16) $$E\;=\;\sum_{k=1}^{n}{\left[u_{1,k}^{\prime }-u_{2,k}^{\prime }\right]}^{2}=\;{\left\|Y_{1}^{\prime }w_{1}-Y_{2}^{\prime }w_{2}\right\|}^{2}$$

with, for sensor i= 1 and 2,

(17) $$w_{i}=(w_{i1},w_{i2},w_{i3}{)}^{T}$$

$$Y_{i}^{\prime }=\left(\begin{array}{c} \begin{array}{ccc} y_{i,2}^{\prime }\; & y_{i,1}^{\prime } & 1\\ \vdots & \vdots & \vdots \\ y_{i,k}^{\prime } & y_{i,k-1}^{\prime } & 1 \end{array}\\ \begin{array}{ccc} \vdots & \vdots & \vdots \\ y_{i,n+1}^{\prime } & y_{i,n}^{\prime } & 1 \end{array} \end{array}\right)$$

Equation (16) can be written in a more compact form as

(18) $$E\;=\;w^{T}Rw$$

with $w={\left(w_{1}^{T},w_{2}^{T}\right)}^{T}$ and

(19) $$R=\left(\begin{array}{cc} Y_{1}{}^{\prime T}Y_{1}{}^{\prime } & -Y_{1}{}^{\prime T}Y_{2}{}^{\prime }\\ -Y_{2}{}^{\prime T}Y_{1}{}^{\prime } & Y_{2}{}^{\prime T}Y_{2}{}^{\prime } \end{array}\right)$$

As w=0 minimizes Equation (18), a constraint on the norm of the filters is added to avoid the trivial zero-solution. The solution minimizing Equation (18) subject to ${\left\|w\right\|}^{2}=1$ is obtained by solving the following eigenvalue problem

(20) $$R\hat{w}=\rho \hat{w}$$

in which $\hat{w}$ is the eigenvector corresponding to the smallest eigenvalue ρ so that from Equations (18) and (20), we have $E={\hat{w}}^{T}R\hat{w}={\hat{w}}^{T}\rho \hat{w}=\rho$.

The deconvolved output $\left(e^{u_{1}^{\prime }}+e^{u_{2}^{\prime }}\right)/2$ is then obtained from $\hat{w}$ and Equation (15). Note however that the unknown source signal u can only be retrieved up to a scaling factor. Also from $\hat{w}$, the sensors’ parameters can be identified with Equation (14).

**Figure 10:**
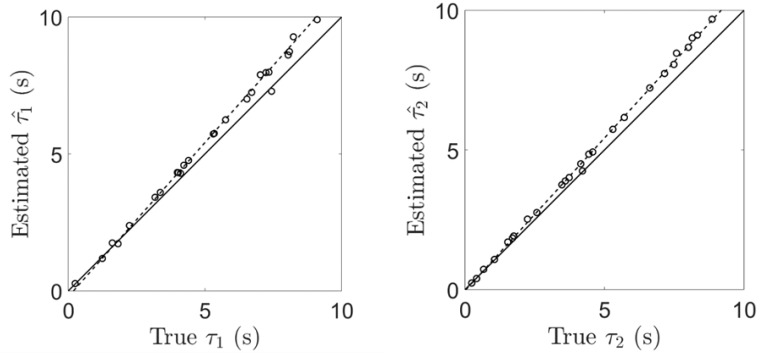
Blind identification of the time constants $\tau_{1}\;$ and $\tau_{2}$ for two simulated MOX sensors. Experiments with simulated MOX sensors (same conditions as in Figure 6). The blind identification method leads to a valid, but biased, estimate: $\hat{\tau_{1}}=1.1\;\tau_{1}$ and $\hat{\tau_{2}}=1.1\;\tau_{2}$.

**Figure 11:**
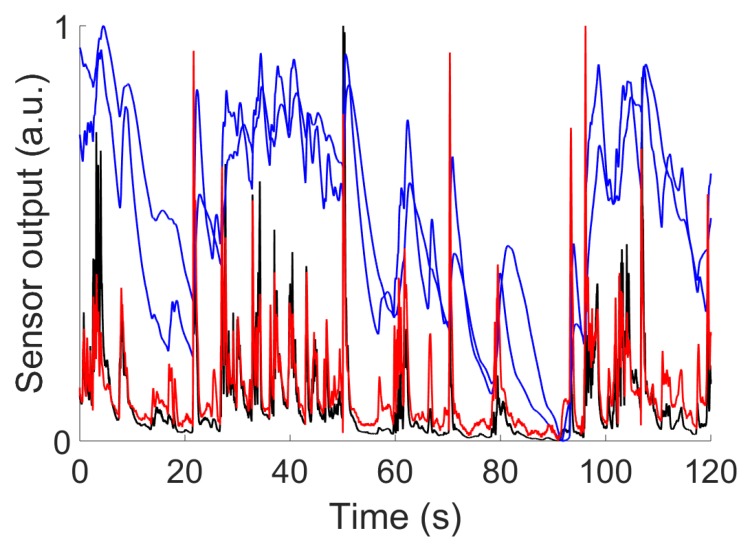
Blind deconvolution, experiments with two real MOX sensors. In blue, the signals $y_{1}$ and $y_{2}$ of the two MOX sensors within an ethanol plume (recording at 15 cm from the releasing source). In red, the signal ($u_{1}$ + $u_{2}$)/2 obtained from the deconvolution of the two MOX sensors. In black, the signal u recorded by the PID at the same location as the MOX sensors. Note that u was not used for the deconvolution (blind) and is shown here for comparison only. Moreover, for comparison, the signals are normalized between 0 and 1 as the blind procedure can only provide u up to a scaling factor.

To validate our blind identification method, we repeated the same experiments as in the previous section but with two MOX sensors. First, when tested it on two simulated MOX sensors. We note in Figure 10 that the blind identification method leads to a valid (although biased) estimate, for the time constants; that is $\hat{\tau }=1.1\;\tau$. Such a blind estimation is possible because the parameter $\hat{a}$ is obtained from Equation (14) via the ratio $-{\hat{w}}_{2}/{\hat{w}}_{1}$ and this ratio is insensitive to the scale ambiguity inherent to the blind procedure. Second, when tested on real data using two MOX sensors, the time constant estimated for the TGS2610 is $\hat{\tau }=2.83$ s with the blind procedure versus 2.47 s with the supervised procedure (see previous section). Yet, these time constants are comparable after compensation of the estimation bias. Figure 11 shows the output $\left(e^{u_{1}^{\prime }}+e^{u_{2}^{\prime }}\right)/2$ obtained after the blind deconvolution of two MOX sensors with Equation 15. We note that the output is similar to the PID signal despite the fact that the PID was not used for deconvolution (blind method). The result obtained is also very close to the one obtained with the related supervised method (compare Figure 11 and Figure 8A).

## 6. Extensions

### 6.1. More Than Two Sensors

Our blind identification method can readily be extended to P>2 sensors as follows. Collecting all possible pairs of sensors gives the cost function

(21) $$E=\sum_{\begin{array}{c} i,j=1\\ i\neq j \end{array}}^{P}{\left\|Y_{i}^{\prime }w_{i}-Y_{j}^{\prime }w_{j}\right\|}^{2}$$

with the $w_{i}$ and $Y_{i}^{\prime }$ defined as in Equation (17). Written in matrix form, Equation (21) reads as follows

(22) $$E\;=\;w^{T}Rw$$

with $w={\left(w_{1}^{T},\cdots ,w_{P}^{T}\right)}^{T}$ and

$$R=\left(\begin{array}{cccc} (P-1)Y_{1}^{\prime T}Y_{1}^{\prime } & -Y_{1}^{\prime T}Y_{2}^{\prime } & \cdots & -Y_{1}^{\prime T}Y_{P}^{\prime }\\ -Y_{2}^{\prime T}Y_{1}^{\prime } & (P-1)Y_{2}^{\prime T}Y_{2}^{\prime } & \cdots & -Y_{2}^{\prime T}Y_{P}^{\prime }\\ \vdots & \ddots & & \\ -{Y^{\prime }}_{P}^{T}Y_{1}^{\prime } & -{Y^{\prime }}_{P}^{T}Y_{2}^{\prime } & \cdots & (P-1)Y_{P}^{\prime T}Y_{P}^{\prime } \end{array}\right)$$

The solution minimizing Equation (22) subject to ${\left\|w\right\|}^{2}=1$ is obtained by solving an eigenvalue problem similar to Equation (20).

### 6.2. Different Time Constants for Rise and Decay

Our deconvolution approach can be extended to a sensor model that switches between two modes of operation with two different time constants depending on whether the sensor conductance increases or decreases. Yet, finding the optimal mode switching as well as the deconvolution filter is a hard combinatorial problem. Thus, we propose a suboptimal procedure in which the switches between the two modes of operation are provided by the slope of the sensor output (rising or decaying conductance). In that case, the deconvolution filter (Equation (9)) writes

(23) $$u_{k}^{\prime }=\{\begin{array}{c} w_{1+}\;y_{k+1}^{\prime }\;+\;w_{2+}\;y_{k}^{\prime }\;+\;w_{3+}\;\mathrm{if}\;\mathrm{mode}+\left(\mathrm{rise}:\;y_{k+1}-y_{k}\geq 0\right)\\ w_{1-}\;y_{k+1}^{\prime }\;+\;w_{2-}\;y_{k}^{\prime }\;+\;w_{3-}\;\mathrm{if}\;\mathrm{mode}-(\mathrm{decay}:\;y_{k+1}-y_{k}<0) \end{array}$$

Let us denote w=($w_{1+},w_{2+},w_{3+},w_{1-},w_{2-},w_{3-}{)}^{T}$ as the *6* *× 1* vector of the concatenated filter coefficients and $Y^{\prime }$ as the extended *n* *× 6* matrix similar to Equation (11), but with entries in the lines of the matrix being either ($y_{k+1}^{\prime },y_{k}^{\prime },1,0,0,0)$ or ($0,0,0,y_{k+1}^{\prime },y_{k}^{\prime },1)$ depending on the mode of operation. Then, Equation (23) can be written in matrix form just as Equation (10)

(24) $$U^{\prime }\;=\;Y^{\prime }\;w$$

Consequently, our least-squares approach also applies to a switching model of MOX sensors and the vector w of filter coefficients can be obtained by the pseudo-inverse solution (Equation (13)).

## 7. Conclusions

In gas plumes, the challenge is to detect intermittent filaments of low concentration. To this aim, the slowness of MOX sensors was compensated by deconvolution based on a non-linear model of MOX sensors. To limit the number of parameters and the noise, we made the model as simple as possible, but not simpler. Our non-linear sensor model contrasts with [4,9]. These previous studies consider a linear model but with two different time constants for the rise and the decay. Despite a single time constant, our model fitted the MOX response reasonably well at low concentration (<100 ppm), see Figure 3B. This may be explained by the fact that our MOX sensors exhibit faster dynamics (due to the miniaturized housing chamber and sensor cap removal) and that the sensor non-linearity is explicitly taken into account in our model.

As shown in the experiments with MOX sensors in turbulent plumes, the model was quite effective in its goals of identification and deconvolution. By applying a logarithmic transformation to the sensor data, we obtained a linearized estimation problem that can be solved efficiently by least-squares techniques. We proposed two practical methods for combined identification/deconvolution: a supervised method that requires prior training with a fast sensor such as a photo-ionization detector and a blind or unsupervised method that exploits merely the information provided by two MOX sensors employed in parallel. One advantage of the blind method is that it allows us to retrain the system on the fly, in the gas plume, in case of sensor replacement or changes in sensor characteristics.

Several lines of research that may prove beneficial and ought to be considered as future work have been detailed in the previous section. These include the extension of our work to multiple sensors and different time constants for rise and decay. The work presented here will certainly foster future applications. Because it allows the tracking of fast-changing gas concentrations, perhaps the most direct implication is in field measurements with olfactory robots or moving vehicles [16]. This task requires fast sensors for two reasons. First, mobile sensing platforms navigate in turbulent environments with a very heterogeneous and discontinuous sensory landscape and, second, when an air quality map is built from measurements acquired in motion, the sensor has to respond quickly or otherwise the resulting map will be blurred [4].

## Figures and Tables

**Figure A1:**
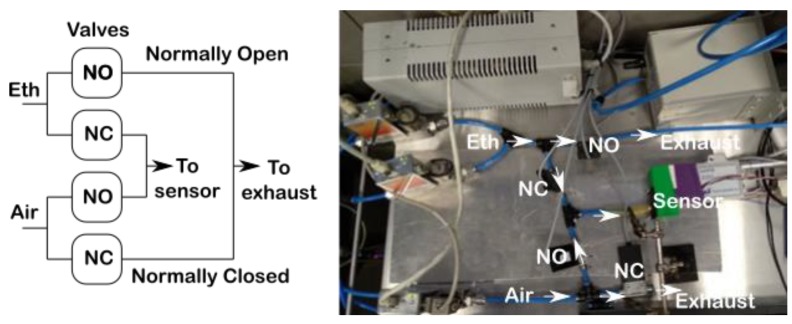
Test bench used in the step experiments. The electrovalves are two-way: normally open (NO) or normally closed (NC). See text in Appendix A for details.

## Appendix A

Step response of MOX sensors and deconvolution. To generate fast concentration steps, we designed a test bench as shown in Figure A1. We used high quality pressurized gases in cylinders (Linde group), mass flow controllers (EL-FLOW Select, Bronkhorst), fast electrovalves (MHJ10-S-2,5-QS-6-HF/LP, Festo Group) with a switching time lower than 1 ms and the necessary fluidic tubes. The two inner valves are mutually exclusive (when one is open the other is closed) so that either synthetic air (99.995% purity and 21 ± 1% O_2_) or ethanol diluted in synthetic air is delivered to the sensors. The two outer valves are used to send the flow that is not being delivered to the sensors (i.e., the one corresponding to the inner valve, which is currently closed) to the exhaust in order to avoid overpressure in the system. The ethanol gas cylinder contained 100 ppm of ethanol diluted in synthetic air with 21 ± 1% O_2_, which was further diluted with synthetic air to reach concentrations below 100 ppm. Three mass flow controllers (MFCs), which had full-scale flow rates of 1000 mL/min, were used to control the rate of dilution of the ethanol gas stream and to fix the flow rate of the final gas stream delivered to the sensors at 1 L/min to match the input flow rate of the pump inside the miniPID.

The response of the MOX to a unit step is modeled with a mono-exponential term as 1−e−tτ for t≥0. The impulse response is given by the derivative of the step response, that is h(t)=1τe−tτ. In response to fluctuating odor concentration u(t), the MOX output is given by the convolution integral x(t)=h(t)∗u(t). Deconvolution is simply obtained by the inverse of the system; that is U(f)=X(f)/H(f) in the frequency domain. Then, U(f)=X(f)+τ2πifX(f) as the Fourier transform of the impulse response is H(f)=1/( 1+τ2πif). Going back in the time domain, the deconvolution filter writes $u\left(t\right)=\;x\left(t\right)+\tau \frac{dx}{dt}$.

For a bi-exponential step response in the form 1−(τ1τ1−τ2e−tτ1−τ2τ1−τ2e−tτ2) for t≥0, it can be shown that the deconvolution in the frequency domain writes $U\left(f\right)=X\left(f\right)+(\tau_{1}-\tau_{2})2\pi ifX\left(f\right)+(\tau_{1}\tau_{2}){\left(2\pi if\right)}^{2}X\left(f\right)$. Thus, in the time domain, the deconvolution filter is $u\left(t\right)=\;x\left(t\right)+(\tau_{1}-\tau_{2})\frac{dx}{dt}+(\tau_{1}\tau_{2})\frac{d^{2}x}{dt^{2}}$.
